# Response to letter regarding “Comparison of lung ultrasound, chest radiographs, C‐reactive protein, and clinical findings in dogs treated for aspiration pneumonia”

**DOI:** 10.1111/jvim.16558

**Published:** 2022-10-03

**Authors:** Nina F. Rodrigues, Léna Giraud, Géraldine Bolen, Aline Fastrès, Cécile Clercx, Søren Boysen, Frédéric Billen, Kris Gommeren

**Affiliations:** ^1^ Department of Clinical Sciences Faculty of Veterinary Medicine, University of Liège Liège Belgium; ^2^ Department of Veterinary Clinical and Diagnostic Sciences University of Calgary Calgary Canada

Dear Editor,

We thank Dr Lisciandro for taking the time to read and write to the editor. However, we believe that Dr Lisciandro has not carefully read the cited references for the lung ultrasound (LUS) protocol used and has therefore incorrectly interpreted the materials and methods of the article. As stated in the materials and methods, the LUS protocol used in the current study was based on modifications to protocols described by Armenise et al in 2019,^1^ and Boysen et al in 2020.^2^ Greater detail of the exact protocol used can be found in the textbook “The Essentials of veterinary point of care ultrasound: Pleural space and lung.”^3^ Therefore sites 1, 6 and 7 in the current study use the sonographically identified caudal‐lateral lung border (the abdominal curtain sign) to guide probe location, ensuring it is never placed over the abdomen as incorrectly implied by Dr Lisicandro. The protocol also uses the scapula and flexor muscles of the shoulder to locate the sonographically definable cranial lung border and extends the limb cranially to allow a larger lung surface area, including the axilla, to be assessed. As referenced in the current study, the pericardio‐diphragmatic window is identified and the probe is turned parallel to the ribs to assess both the ventral pleural and ventral lung regions (sites 3, 4, 9).^2^ The probe is therefore never placed over the flexor muscles of the shoulder or scapula as erroneously concluded by Dr Lisciandro. For logistical reasons, cineloops were taken at the intercostal sites listed in the paper, but more lung surface was assessed in the protocol than cineloops recorded for comparison to radiographs, which might be contributing to some of the confusion expressed by Dr Lisciandro. The sites used for comparison to thoracic radiographs were selected sites from a larger LUS protocol and are therefore the correct sites as cited in the paper. Although we did not record a cineloop at the axilla region for comparison with radiographs, it was assessed in the current and other published LUS protocols,^2^, ^3^ making Dr Lisciandro's statement that VetBLUE is the only lung ultrasound protocol to assess the axilla region incorrect. By using sonographically defined borders the operator is able to ensure lung is assessed, and by extending the limb cranially sites 3 and 4 are easily assessed bilaterally.

Although we used sonographically defined lung borders to standardize the protocol between animals, we should point out that because of the “dome‐shape” of the diaphragm, and the anatomic and physiologic interaction of the lung, diaphragm and costophrenic recesses, the cranial outline of the diaphragm on a lateral radiograph does not equate to the caudal margins of the lung. This is because the lung overlies and extends over the dome‐shaped diaphragm and soft tissues of the abdomen along the costophrenic recess, giving rise to a vertical edge artifact referred to as the “abdominal curtain sign.”^3^ In the authors' experience, this border tends to vary with species, breed, respiratory effort and underlying lung lesions.^3^ It is also the authors' experience that it can be difficult to determine the caudal extent of the lung margin on a lateral radiograph, particularly given this border changes during the respiratory cycle. We believe it is therefore misleading to conclude that a region located just caudal to the visible cranial edge of the diaphragm on a lateral radiograph, particularly in the mid to upper thoracic regions, will equate to the abdominal contents of the abdomen when assessed with ultrasound. This is because ultrasound can only assess the surface of aerated lung and cannot penetrate more than 1 to 2 mm in depth from the surface. Therefore, although soft tissues are visible medial to overlying lung on a lateral radiograph, it is not possible to see them when using ultrasound. The variation in the caudal lung margins with respiratory cycle and effort, species, breed, and underlying lung pathology is why a LUS protocol that uses sonographically defined lung borders is preferred by the authors and was used in the current study.

It is the authors' experience that the LUS protocol used in the current study allows more precise identification of the most caudo‐dorsal pleural and lung ultrasound site (the starting point) than many LUS protocols, including VetBLUE, because it locates this site using sonographically defined borders.^2^, ^3^ This is in contrast to less precise descriptive terms such as “the chest tube site,” which may vary depending on why a chest tube is placed (pleural effusion vs pneumothorax) and clinician preference.^3^ Although an effort to standardize VetBLUE within veterinary medicine has been attempted, several recent papers authored by Dr Lisciandro and others assess only a single intercostal space at each of the 4 bilaterally examined sites of the thorax,^4^ while others list 3 or possibly more intercostal spaces/per thoracic site examined,^5^ or fail to define the number of intercostal sites assessed. This makes comparison of results between studies challenging. Finally, it is interesting to note that as VetBLUE increases the sites assessed from a single intercostal space to multiple intercostal spaces, it more closely aligns with earlier studies published by Dr Armenise^6^ and the protocol used in the current study. The main difference being that the protocol published by Dr Armenise assesses more intercostal spaces than currently published VetBLUE protocols. Although LUS comparison studies are extremely limited in veterinary medicine, there is preliminary evidence to suggest the incidence of B‐lines is higher in protocols that scan larger lung surface areas in cats, dogs and humans,^7^ however this may or may not translate to pathologic conditions. An abstract in dogs suggests LUS protocols that examine larger lung surface area can detect pathology otherwise missed with protocols that scan less lung surface area, although this is a small study and prospective veterinary studies are needed to know how many sites need to be scanned to maximize sensitivity and specificity at finding underlying pleural and lung pathology.^8^ The duration of time to perform lung ultrasound will likely need to be balanced against the speed with which a diagnosis needs to be made.

We hope that clarifies things and once again thank Dr Lisicandro for his comments, and for providing us with the opportunity to further clarify the protocol used in the current study.
